# An equivalent realization of coherent perfect absorption under single beam illumination

**DOI:** 10.1038/srep07369

**Published:** 2014-12-08

**Authors:** Sucheng Li, Jie Luo, Shahzad Anwar, Shuo Li, Weixin Lu, Zhi Hong Hang, Yun Lai, Bo Hou, Mingrong Shen, Chinhua Wang

**Affiliations:** 1 College of Physics, Optoelectronics and Energy, Soochow University, Suzhou 215006, China; 2 Jiangsu Key Lab of Advanced Optical Manufacturing Technologies & Collaborative Innovation Center of Suzhou Nano Science and Technology, Soochow University, Suzhou 215006, China

## Abstract

We have experimentally and numerically demonstrated that the coherent perfect absorption (CPA) can equivalently be accomplished under single beam illumination. Instead of using the counter-propagating coherent dual beams, we introduce a perfect magnetic conductor (PMC) surface as a mirror boundary to the CPA configuration. Such a PMC surface can practically be embodied, utilizing high impedance surfaces, i.e., mushroom structures. By covering them with an ultrathin conductive film of sheet resistance 377 Ω, the perfect (100%) microwave absorption is achieved when the film is illuminated by a single beam from one side. Employing the PMC boundary reduces the coherence requirement in the original CPA setup, though the present implementation is limited to the single frequency or narrow band operation. Our work proposes an equivalent way to realize the CPA under the single beam illumination, and might have applications in engineering absorbent materials.

Recently, coherent perfect absorption (CPA) has been proposed as a new concept of accomplishing 100% perfect absorption, and has attracted a lot of research interest spanning from optics to acoustics^1^,^2^,^3^,^4^,^5^,^6^,^7^,^8^,^9^,^10^,^11^,^12^,^13^,^14^,^15^,^16^,^17^,^18^,^19^,^20^,^21^,^22^. The proposed CPA phenomenon takes place inside a dielectric Fabry-Perot (FP) cavity when it is illuminated by two coherent counter-propagating light beams. At the absorption frequency, all input electromagnetic (EM) energy is trapped and dissipated inside the cavity, giving rise to 100% absorption. From the optical resonant cavity configuration, the original CPA operates to a specific frequency and the wavelength thick material^2^. In the past few years, the investigations on the CPA phenomenon have been extended to various material systems including plasmonics^3^,^4^, complex media^5^,^10^, photonic crystals^11^,^12^,^13^, graphene photonics^14^,^15^, etc. and to different research fields, for instance, terahertz optics^16^, nonlinear optics^17^,^18^, and acoustic waves^19^,^20^. Very recently, using the ultrathin conductive film the CPA can be observed in radio frequency and microwave domain, and may exhibit the unprecedented absorption bandwidth and extremely subwavelength thickness advantages^6^,^21^,^22^. However, in many practical applications, perfect absorption under single beam illumination has a greater benefit than the illumination of two opposite beams which have to satisfy the coherence condition. In the previous absorption study, the critical coupling mechanism can lead to perfect absorption at the single beam case^23^,^24^,^25^, and has been employed for resonance enhanced photodetectors^26^,^27^. Also, the critical coupling condition holds usually at a single frequency or some discrete frequencies, and the latter case can be engineered to exhibit a relatively broadband absorption if material components are also broadband.

Metamaterials, through varying the size and shape of subwavelength metallic elements, can provide nearly arbitrary control over the electric and magnetic response of materials. Therefore, metamaterials have exhibited exotic EM phenomena including artificial magnetism, negative refraction, superlens, invisibility cloaks, etc^28^,^29^,^30^,^31^,^32^., and have enabled novel device applications, for instance, metasurfaces^33^,^34^,^35^,^36^ and perfect absorbers^37^,^38^,^39^,^40^,^41^,^42^,^43^,^44^,^45^,^46^,^47^. In particular, a type of high-impedance surfaces (HIS's) which is made of mushroom-like elements on a ground plate is conceived to provide the zero-phase reflection, and thereby mimics a perfect magnetic conductor (PMC) boundary^48^,^49^. The functionality of the HIS is a consequence of the magnetic resonance localized between the mushroom element and the ground plate, and is easily tailored by the mushroom size, the structure periodicity and the dielectric constant.

In this study, we numerically and experimentally demonstrate that the CPA can equivalently be realized under single beam illumination by introducing the PMC boundary. Such boundary behaves like a mirror and mitigates the incident condition of coherent dual beams. In experiments, we have employed the HIS to perform the PMC boundary, and observed the complete EM absorption when a single beam of microwave impinges on the HIS-backed ultrathin conductive film.

## Results

**Figure 1:**
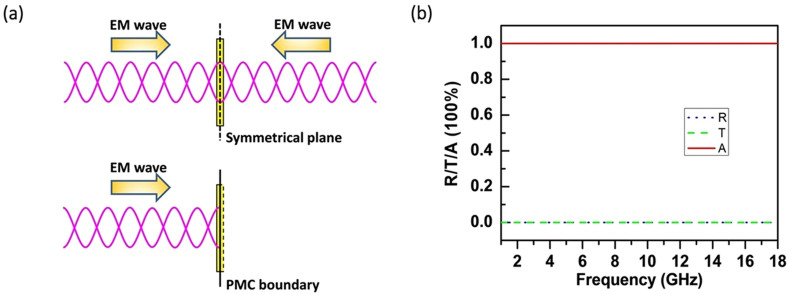
The equivalent realization of CPA under single beam illumination. (a) The schematic drawings of the symmetrical dual beam CPA (upper panel) and the PMC boundary implementation (lower panel). The symmetrical plane (dash line) is the interfering position with the same optical paths to the microwave source for two counter-propagating beams. (b) The calculated reflectance, transmittance, and absorbance of the conductive film, *R_s_* = 377 Ω, supported by the PMC boundary in microwave frequencies, 1–18 GHz.

As demonstrated by the thin film CPA experiments in microwave regime^21^, the frequency-independent perfect absorption occurs at the ultrathin conductive film which is illuminated by two opposite plane waves with the same amplitude, phase, and polarization. The standing wave is established by the two counter-propagating coherent beams which may come experimentally from two identical horn antennas connected with a common source via microwave power dividers/splitters. In a symmetrical arrangement, the constructive interference between the two beams with zero-path delay takes place at the film position, i.e., the symmetrical plane which is the same distant to the microwave source^21^, which is schematically shown in Fig. 1(a). From the field boundary condition point of view, the electric field (E-field) is enforced whereas the magnetic field is cancelled at the symmetrical plane to every EM wavelength, which is just the consequence of the zero-path delay interference between the two counter-propagating beams and is regarded as one key prerequisite to the frequency-independent microwave CPA. Another crucial requirement is the ultrathin profile of the film which excludes the wavelength-dependent FP cavity resonance. When the ultrathin conductive film is located, the enforced E-field as well as the associated EM energy is finally dissipated as ohmic loss inside the film via the free carrier absorption. The simple calculation reveals that the complete annihilation of two beams of EM waves happens when the sheet resistance, *R_s_* (also called square resistance), of the film is one half of the vacuum impedance, Z_0_^21^. Alternatively, we may obtain such field boundary condition by employing an ideal PMC surface where the parallel component of the E-field is allowed and the parallel component of the H-field is forbidden. Also illustrated in Fig. 1(a), a PMC surface is inserted at the position which plays the same role as the symmetrical plane of the CPA configuration, and performs like a mirror with the zero-phase, 100% reflection, which thereby gives rise to the same standing wave pattern as the CPA but only for the left half space. In addition, the full band performance of the ideal PMC guarantees such field boundary emerging at each frequency. Such mirror effect rendered by the PMC surface is equivalent to launching another coherent microwave beam from the right side, and has been explored in the antenna radiation engineering and the optoelectronic device designs^50^,^51^.

The sample is an ultrathin conductive film leaning on the PMC mirror. In order to achieve the perfect absorption in this situation, the sheet resistance is modified to be *R_s_* = 1/*σh* = Z_0_, where the conductivity *σ* can be considered nondispersive in radio frequencies and microwave regime, *h* is the film thickness, and Z_0_ = 377 Ω. The sheet resistance condition can be easily understood following the previous frequency-independent thin film CPA condition *R_s_* = Z_0_/2. Compared with the symmetrical configuration under two beam illumination, the film is bisected by the PMC now, and consequently its thickness is reduced by one half, and hence the sheet resistance will become twice times Z_0_/2. The relevant calculation can confirm this perfect absorption condition, too^22^. Here, we have used the finite-element-method simulation software, COMSOL Multiphysics, to investigate numerically the single beam case and the resistance condition. Utilizing a hypothetical PMC boundary over the whole EM spectra in the simulation and the resistive film of *R_s_* = 377 Ω, we obtained the reflectance, transmittance and absorbance, as plotted in Fig. 1(b). It is seen that the perfect absorption can be achieved in a frequency-independent manner, which is equivalent exactly to the symmetrical CPA.

This equivalent realization of CPA, based on the PMC mirror, is noticed to be similar to the aforementioned critical coupling effect in aspects of the thin absorbent layer and the single beam operation. However, the obvious distinction of the former from the latter lies in the peculiar zero-phase unitary reflector which is the most appealing property of the ideal PMC mirror and enables the ultrathin profile benefit and the ultra-broadband applications. Therefore, there is the nice correspondence between the PMC based perfection absorption and the dual beam CPA with zero-path delay interfering system. In contrast, the typical configuration for the critical coupling absorbers involves the dielectric mirror (e.g., Bragg mirror or chirped mirror) which is a reflector with a couple of wavelengths thickness and band performance. Because of its multiple partial reflection principle, the dielectric mirror fails to generate the intuitive physical picture corresponding to the CPA of zero-path delay interference.

It is well known that the ideal PMC doesn't exist in practice. Although several artificial PMC schemes have been verified, for example, the mushroom HIS^48^, the grooved metal^51^, and the epsilon-near-zero media^52^, all the PMC responses are limited to the specific frequency.

**Figure 2:**
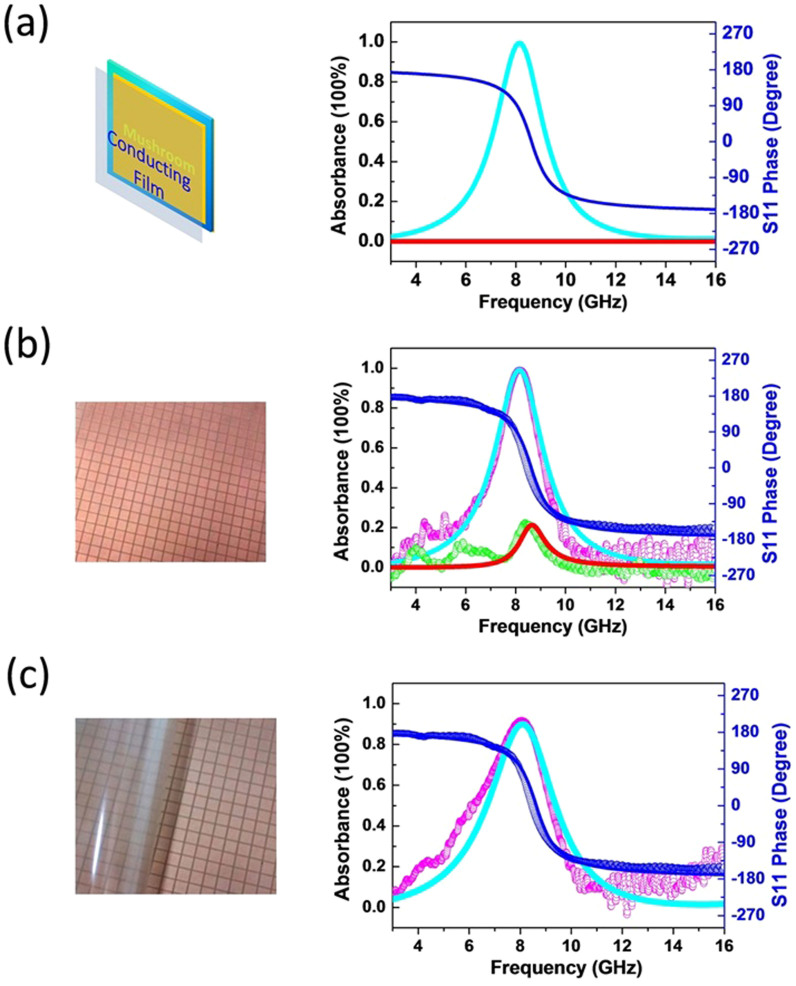
The calculated and measured absorbance and reflection phase. (a) Left: the schematic drawing of the unit cell. Right: the calculated absorbance (red solid line) and the S11 phase (blue solid line) of the ideal mushroom structure and the calculated absorbance of the composite structure with the conductive film of *Rs* = 377 Ω (cyan solid line). (b) Left: the photo of the mushroom sample. Right: the measured absorbance (green symbols) and the S11 phase (blue symbols) of the mushroom sample and the measured absorbance of the composite structure with the conductive film of *Rs* = 383 Ω (pink symbols). The solid lines are the corresponding simulated results. (c) Left: the photo of the sample with the peeled conductive film. Right: the measured (pink symbols) and simulated (cyan solid line) absorbance of the composite structure with the conductive film of *Rs* = 214 Ω.

In our experimental configuration, the PMC surface is practically carried out by utilizing the HIS with mushroom structure for one or more specific working frequencies. The mushroom HIS is constructed of a periodic array of square metallic patches printed on a dielectric layer backed by a ground plane. The square patches of side length *a* = 3 *mm* are arranged into a square lattice with periodicity *p* = 3.3 *mm*, as shown in Figs. 2(a) and 2(b). Here, in the first place, we numerically reveal that the perfect absorption is caused only by the ultrathin conductive film. In Fig. 2(a), the red solid line is the simulated absorption spectrum of an ideal mushroom structure where all metallic parts are approximated as the perfect electric conductor and the dielectric layer is lossless in the numerical model. It is very obvious that the mushroom HIS does not absorb any microwaves. Furthermore, from the S11 phase spectrum, we find a zero reflection phase at about 8.6 GHz, which displays the property of PMC at this specific frequency. Then, we attach an ultrathin conductive film of *R_s_* = 377 Ω in front of the mushroom patches and compute the absorption of the composite structure. As shown as cyan solid line in Fig. 2(a), perfect absorption is obtained at about 8.2 GHz. The small frequency shift with respect to the zero phase position originates from the mushroom microstructure of the HIS. Due to the local resonance in the microstructure, the waves are not ideal plane waves at the region close to the HIS, even though they become good plane waves in the far-field region.

The HIS sample was fabricated on a standard printed circuit board (PCB) with a thickness of 0.8 mm. The dielectric layer has the relative permittivity 13.8 and is coated by the 18 um copper claddings on both faces. After patterning the mushroom patches on one face, we measured and calculated the reflection of the HIS. In the absorption measurement, we have employed a microwave network analyzer (Agilent N5230C) and connected two rectangular horn antennas with port 1 and 2 of the network analyzer. The transmission magnitude and phase is determined by the S21 signal with the sample in presence normalized with respect to the signal without the sample. The reflection magnitude and phase is determined by the S11 signal of the sample normalized with respect to the signal of a copper plate. Finally, the absorbance, *A*, is calculated as 1−|S11|^2^−|S21|^2^, where |S11|^2^ represents reflectance and |S21|^2^ transmittance. However, because of the metal ground plane in the HIS structure, the transmittance is found being zero. Thus, the absorbance, *A*, is calculated as 1 − |S11|^2^ simply.

As plotted in Fig. 2(b), the zero-phase reflection of the HIS is identified at ~8.6 GHz, agreeing well with the simulation. However, a tiny absorption peak at the corresponding zero-phase reflection frequency is seen, due to the inevitable dielectric loss as well as the metallic loss in the PCB. Then, we place an ultrathin conductive film on top of the mushroom sample. The conductive films are just transparent conductors which are widely used in optoelectronic and consumer electronic devices^53^. The experimental film comes from the customized commercial touch screen product (SVG Optronics), which has the 0.2 mm thick flexible plastic substrate and the 2.6 um thick conducting layer. Note that the conducting layer thickness, i.e., the dissipating thickness, is only ~λ/10000 (λ denoting the wavelength in vacuum). The sheet resistance of the film was 383 Ω, characterized by the four-probe instrument. No bonding paste is applied between the conductive film and the mushroom patches, and hence the former is completely peelable from the mushroom surface, seeing the photo in Fig. 2(c). Apparently, the almost perfect absorption can be observed at about 8.2 GHz in Fig. 2(b), and the measured results are seen to coincide very well with the simulated ones. The film sample of *R_s_* = 214 Ω is also simulated and measured in our work, and the results are plotted in Fig. 2(c). It should be noted that the maximal absorption is only about 90%, which justifies the aforementioned resistance condition for the perfect absorption.

**Figure 3:**
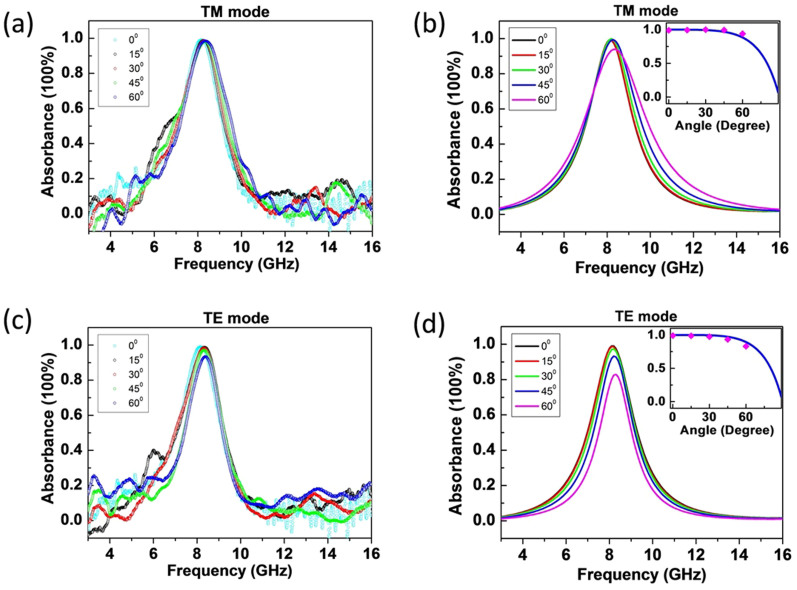
The absorbance of the composite structure with the conductive film of *R*_s_ = 383 Ω under off-normal incidence. (a)–(d) The measured and calculated absorbance under TM and TE modes. The angular variations of the TM (TE) absorbance (vertical axis) are illustrated, respectively, as the insets where the symbols denote the calculated maximum absorbance in (b) and (d) and the line is the angular absorption of the dual beam CPA for the conductive film of *R*_s_ = 191.5 Ω.

Similar to the thin film CPA phenomenon, the composite structure combining the conductive film and the HIS also support the nearly perfect absorption in the off-normal incidence. In measurement, four incident angles, namely, *θ* = 15°, 30°, 45°, 60°, are selected. The measured results for the composite absorber with sheet resistance *R*_s_ = 383 Ω are depicted in Figs. 3(a) and 3(c). It is seen that larger than 90% TM&TE maximum absorbance can be obtained experimentally even under the 60 degree oblique incidence, which indicates a very large angular tolerance. The calculation in Figs. 3(b) and 3(d) show good agreement with the measurement. In particular, as illustrated by the insets, the angular variation of the absorbance at the peak frequency is consistent with the dual beam CPA case of the ultrathin conductive film under oblique illumination (the lines in the insets given by *A* = 1 − |(*R_s_* − 0.5*Z*_0_cos*θ*)/(*R_s_* + 0.5*Z*_0_cos*θ*)|^2^ for TM mode and *A* = 1 − |(*R_s_* − 0.5*Z*_0_/cos*θ*)/(*R_s_* + 0.5*Z*_0_/cos*θ*)|^2^ for TE mode, both with *R*_s_ = 191.5 Ω, seeing Refs. 21, 22).

**Figure 4:**
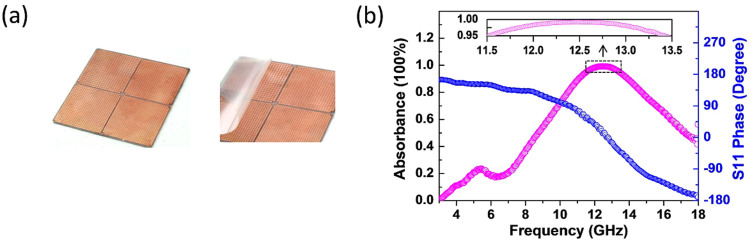
The sample composed of four different sizes of mushroom patches. (a) Photo of the sample without and with the peelable conductive film. (b) The measured absorption (magenta open symbols) of the composite structure with the conductive film of *R*_s_ = 383 Ω and the S11 phase (blue open symbols) of the bare HIS. The inset shows the frequency band with absorbance greater than 95%.

Because of the resonance nature of the HIS, the PMC property, i.e. the zero-phase reflection, appears only at single frequency. A numerous efforts of developing the HIS with band performance have been initiated across the whole EM spectrum from microwave to visible. However, it is quite challenging to conceive a broadband artificial PMC, of which the reflection phase is pinned to zero over a band of frequencies. A practically effective way of improving the bandwidth in most designs is to integrate multiple sized mushroom elements on the single HIS^54^,^55^. In this way, we have fabricated a HIS which consists of four regions of the mushroom patches with different sizes, as shown in Fig. 4(a). Their sizes are *p*_1_ = 3.3 *mm*, *a*_1_ = 3 *mm*, *p*_2_ = 3 *mm*, *a*_2_ = 2.8 *mm*, *p*_3_ = 2.7 *mm*, *a*_3_ = 2.5 *mm*, *p*_4_ = 2.5 *mm*, *a*_4_ = 2.3 *mm*. In Fig. 4(b), we measured the S11 phase of the bare HIS and the normally incident absorption of the HIS covered with the conductive film of *R*_s_ = 383 Ω. The band of the in-phase reflection of the HIS can be loosely defined as frequency range, Δ*f*, with the phase decreasing continuously from +90° to −90°, i.e., Δ*f* = |*f*(+90°) − *f*(−90°)|, where *f*(+90°) and *f*(−90°) denote the frequencies of the +90° and −90° reflection phase, respectively^48^. Corresponding to a larger bandwidth Δ*f* = 3.95 GHz for the multiple sized patch sample, the dispersive slope of the phase curve around zero level in Fig. 4 is seen less than that in Fig. 2 where Δ*f* = 1.16 GHz. So the absorption peak of the composite structure becomes plateau-like and the frequency band with absorbance over some level, say >95%, is expanded, as illustrated by the inset in Fig. 4(b). Also, the >50%(3 dB) absorption is noticed to range from 8.7 to 17.2 GHz. Although the band broadening can be achieved, to some degree, via varying the patch size, it is not a solution of broadband PMC in a rigorous sense (the reflection phase keeping zero within a wide band). Additionally, the varying gradient and dispersing topology of different sized patches will influent intensively the absorption perfectness and the bandwidth.

## Discussion

From the practical limitation of the HIS structure, the equivalent CPA of the ultrathin conductive film occurs only to single frequency or narrow band, though the illumination condition relaxes to the single beam. The use of the resonant HIS, rather than the ideal PMC, results in the spectral characteristic is similar to those of the metamaterial absorbers which usually rely on resonance^37^,^38^,^39^,^40^,^41^,^42^,^43^,^44^,^45^,^46^,^47^, due to missing the distinctive frequency-independency. Therefore, the development of an ultra-broadband, even frequency-independent, PMC in the future will be much beneficial to our study. In addition, the structural separation of the substrate layer and the dissipative layer (the conductive film) in our case is different from the usual design of metamaterial absorbers where the supporting dielectric substrate often serves as the major absorbing component^37^. This separating layout leads to the angular performance of the absorption in our case is governed, to a large extent, by the overlying conductive film. It is seen that the angular absorption in the insets of Fig. 3 is consistent with the dual beam CPA picture.

Employing the soft transparent conductor as the detachable resistive layer for the EM absorption will bring about more designing flexibilities, such as conformality over curved substrates and mechanical peelability, than the traditional HIS-based metamaterial absorbers which need load a series of lumped resistors and/or embed the prescribed lossy dielectrics^56^,^57^,^58^. The transparent conductor layer owns the aesthetical effect of optical transparency and suits the application need of transparent absorbers^59^. In a context of transparent conductors being broadly integrated in various optoelectronic/electronic devices, our results might also stimulate a general interest of exploring the electromagnetic absorbing/shielding in the developing ultrathin, light-weight, mobile devices where the flexible transparent conductors are crucial parts^60^.

In conclusion, the CPA can equivalently be realized under single beam illumination by using a PMC surface to meet the same prerequisite of the field components as under two beam illumination. Consequently, the perfect absorption condition changes from *R*_s_ = Z_0_/2 to *R*_s_ = Z_0_. In addition, the angular tolerance at the oblique TM&TE incidence and the improvement of broadening the absorption band are explored. Such an equivalent implementation breaks the constraint of two coherent incident beams with the identical amplitude and phase, and might have applications in engineering absorbent materials.

## Author Contributions

S.L. did the experiments and calculations; J.L. and Y.L. suggested the research; S.A. and S.L. assisted the measurements; W.L., Z.H.H., Y.L. and B.H. analyzed the data; M.S. and C.W. customized the samples; B.H. supervised the project and wrote the manuscript.
